# Ultra-monochromatic far-infrared Cherenkov diffraction radiation in a super-radiant regime

**DOI:** 10.1038/s41598-020-76996-1

**Published:** 2020-12-01

**Authors:** P. Karataev, K. Fedorov, G. Naumenko, K. Popov, A. Potylitsyn, A. Vukolov

**Affiliations:** 1 John Adams Institute at Royal Holloway, University of London, Egham, TW20 0EX Surrey UK; 2 National Research Tomsk Polytechnic University, Lenin Ave. 30, 634050 Tomsk, Russia

**Keywords:** Infrared spectroscopy, Terahertz optics, Microwave photonics, Free-electron lasers, Frequency combs

## Abstract

Nowadays, intense electromagnetic (EM) radiation in the far-infrared (FIR) spectral range is an advanced tool for scientific research in biology, chemistry, and material science because many materials leave signatures in the radiation spectrum. Narrow-band spectral lines enable researchers to investigate the matter response in greater detail. The generation of highly monochromatic variable frequency FIR radiation has therefore become a broad area of research. High energy electron beams consisting of a long train of dense bunches of particles provide a super-radiant regime and can generate intense highly monochromatic radiation due to coherent emission in the spectral range from a few GHz to potentially a few THz. We employed novel coherent Cherenkov diffraction radiation (ChDR) as a generation mechanism. This effect occurs when a fast charged particle moves in the vicinity of and parallel to a dielectric interface. Two key features of the ChDR phenomenon are its non-invasive nature and its photon yield being proportional to the length of the radiator. The bunched structure of the very long electron beam produced spectral lines that were observed to have frequencies upto 21 GHz and with a relative bandwidth of 10^–4^ ~ 10^–5^. The line bandwidth and intensity are defined by the shape and length of the bunch train. A compact linear accelerator can be utilized to control the resonant wavelength by adjusting the bunch sequence frequency.

## Introduction

A fast particle passing by an atom interacts with the electron shell forming a dipole that oscillates, inducing polarization currents that are changing in time^1^. Those currents give rise to an entire family of polarization radiation mechanisms that have been widely applied for both charged particle beam diagnostics and the generation of intense EM radiation beams, e.g. transition radiation^2,3^, diffraction radiation^4,5^, Smith-Purcell radiation^6–9^, and parametric X-ray radiation^10^. More recently it has been proposed to use plasmons generated by the field of passing particle in a thin conducting material to reinforce polarization radiation photon yield at well-defined frequencies^11,12^.

Classic Cherenkov Radiation (ChR) is a member of the polarization radiation family and appears whenever a fast charge particle moves in a medium with the speed higher than the speed of light inside that medium. In 1934 it has been discovered experimentally by Pavel Cherenkov^13^, who was awarded the Nobel Prize in 1958. The radiation propagates radially away from the particle trajectory at a classical Cherenkov angle defined as:

1 $$cos\left( {\theta_{{{\text{ch}}}} } \right) = \frac{{v_{photon} }}{{v_{electron} }} = \frac{1}{\beta n},$$

where *v*_*photon*_ = *c/n* is the speed of light inside the medium, *β* = *v/c* is the speed of the particle, *v* = *v*_*electron*_, in units of the speed of light in vacuum, *c*, and *n* is the refractive index. Tamm and Frank have developed a theoretical approach to describe the classical ChR characteristics^14^. Recently more advanced ChR mechanisms have been considered for intense radiation generation including excitation of structural materials such as metamaterials^15^, gratings^8,9,16^, or photonic crystals^17,18^.

**Figure 1:**
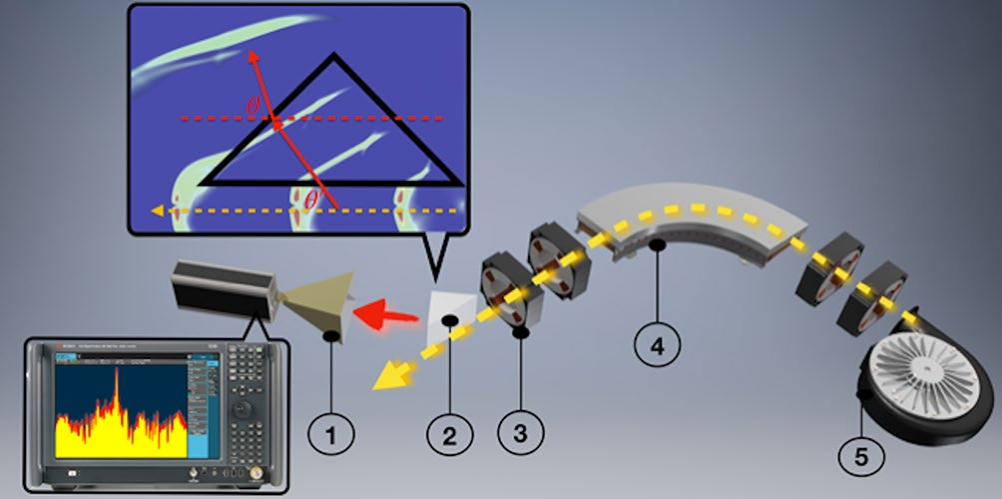
Schematic layout of the experimental installation. The pre-bunched 6.1 MeV electron beam is generated in a microtron accelerator **(5)** (see “Methods”). A dipole magnet **(4)** directs the beam into the experimental area. Two quadrupole magnet doublets **(3)** shape the beam in the vicinity of the target **(2)**. The target itself is a Teflon triangular prism made such that its output interface is normal to the Cherenkov direction to minimize the refraction deflection shown in the top insertion. The radiation extracted from the target is fed into a multi-octave horn-antenna **(1)** (see “Methods”). The radiation field induced currents were fed into a high-speed spectrum analyser (see “Methods”) which monitors the radiation spectrum from every train shot-by-shot.

For a considerably long time it was assumed that a charged particle must physically interact with the medium to generate ChR photons. Modern accelerator facilities produce ultra-relativistic electrons. Any medium will be polarised if it is placed at an impact parameter (*h –* the shortest distance between the particle trajectory and the medium^19,20^) smaller than the effective ultra-relativistic electron field radius defined as

2 $$h \le \frac{\gamma \lambda }{{2\pi }},$$

where *γ* is the charged particle Lorentz-factor and *λ* is the radiation wavelength. This parameter can reach macroscopic values, e.g. for *γ* = 12 and *λ* = 28.5 mm, any object placed within 54 mm will interact with the charged particle field. If a fast charged particle moves in the vicinity of and parallel to a dielectric interface, its field will polarize every atom along the medium surface generating photons propagating into the medium (Fig. 1 top insertion). Since the particle speed is higher than the speed of light inside the medium, the radiation propagates at a classical Cherenkov angle defined by the ratio of the velocities resulting in Eq. (1). The spectral-angular properties of the radiation are significantly different from classical ChR, e.g. the finite boundaries of the radiator cause diffraction, narrowing down the radiation cone and the non-zero impact parameter introduces additional frequency dependence^19,20^. Therefore, a new name was introduced – Cherenkov Diffraction Radiation (ChDR)^19^. Incoherent ChDR, which is a linear function in relation to the number of electrons in a single bunch, has recently been directly observed at the University of Cornell^19^. This experiment has demonstrated fundamental features of the ChDR phenomenon including high directionality in a narrow solid angle and an exponential decrease of intensity as a function of the impact parameter.

Coherent radiation is generated at a wavelength comparable to or larger than a single bunch length^21^, i.e. when all electrons emit radiation more or less in phase. The emission of radiation in the presence of the field from neighbouring particles results in coherent enhancement, which makes the intensity be proportional to the square of the bunch population.

A super-radiant emission appears when a train of equally spaced bunches emits radiation coherently. Assuming a train moving in the vicinity of and parallel to an interface with known dielectric properties as is shown in Fig. 1, a general expression for radiation intensity is^21,22^

3 $$\frac{{dW_{{{\text{train}}}} }}{d\omega } = N_{{\text{e}}}^{2} \frac{{dW_{{\text{e}}} }}{d\omega }F_{{\text{b}}} \left( {\omega ,\sigma } \right)G_{{{\text{train}}}} \left( {\omega ,f_{{\text{b}}} } \right),$$

where *N*_e_ is the number of particles in a single bunch, *ω* = *2πc/λ* is the photon frequency, *f*_b_ is the bunch sequence frequency, *σ* is the rms longitudinal beam size, *dW*_e_/*dω* is the spectrum generated by a single particle (for calculations we used the model derived by Konkov and Shevelev^24^, see “Methods”).

The longitudinal bunch form factor, *F*_b_, is defined as a Fourier transform of the longitudinal particle distribution, *ρ*, in a bunch^21^. For a Gaussian profile

4 $$F_{{\text{b}}} \left( {\omega ,\sigma } \right) = \left| {\mathop \smallint \limits_{ - \infty }^{\infty } \rho \left( {z,\sigma } \right)e^{{ - i\frac{\omega z}{c}}} dz} \right|^{2} = e^{{ - \frac{{\sigma^{2} \omega^{2} }}{{c^{2} }}}} .$$

The train form factor, *G*_train_, is defined as a superposition of the radiation fields generated by a series of consecutive bunches. Assuming that each bunch out of *N*_b_ bunches generates the same amplitude, the train form factor is^22,23^

5 $$\begin{aligned} G_{{{\text{train}}}} \left( {\omega ,f_{{\text{b}}} } \right) & = \left| {1 + e^{{ - i\frac{\omega }{{f_{{\text{b}}} }}}} + e^{{ - i\frac{2\omega }{{f_{{\text{b}}} }}}} + \ldots } \right|^{2} \\ & = \left| {\frac{{1 - e^{{ - i\frac{{N_{{\text{b}}} \omega }}{{f_{{\text{b}}} }}}} }}{{1 - e^{{ - i\frac{\omega }{{f_{{\text{b}}} }}}} }}} \right|^{2} = \frac{{sin^{2} \left( {\frac{{N_{{\text{b}}} \omega }}{{2f_{{\text{b}}} }}} \right)}}{{sin^{2} \left( {\frac{\omega }{{2f_{{\text{b}}} }}} \right)}} \\ \end{aligned}$$

**Figure 2:**
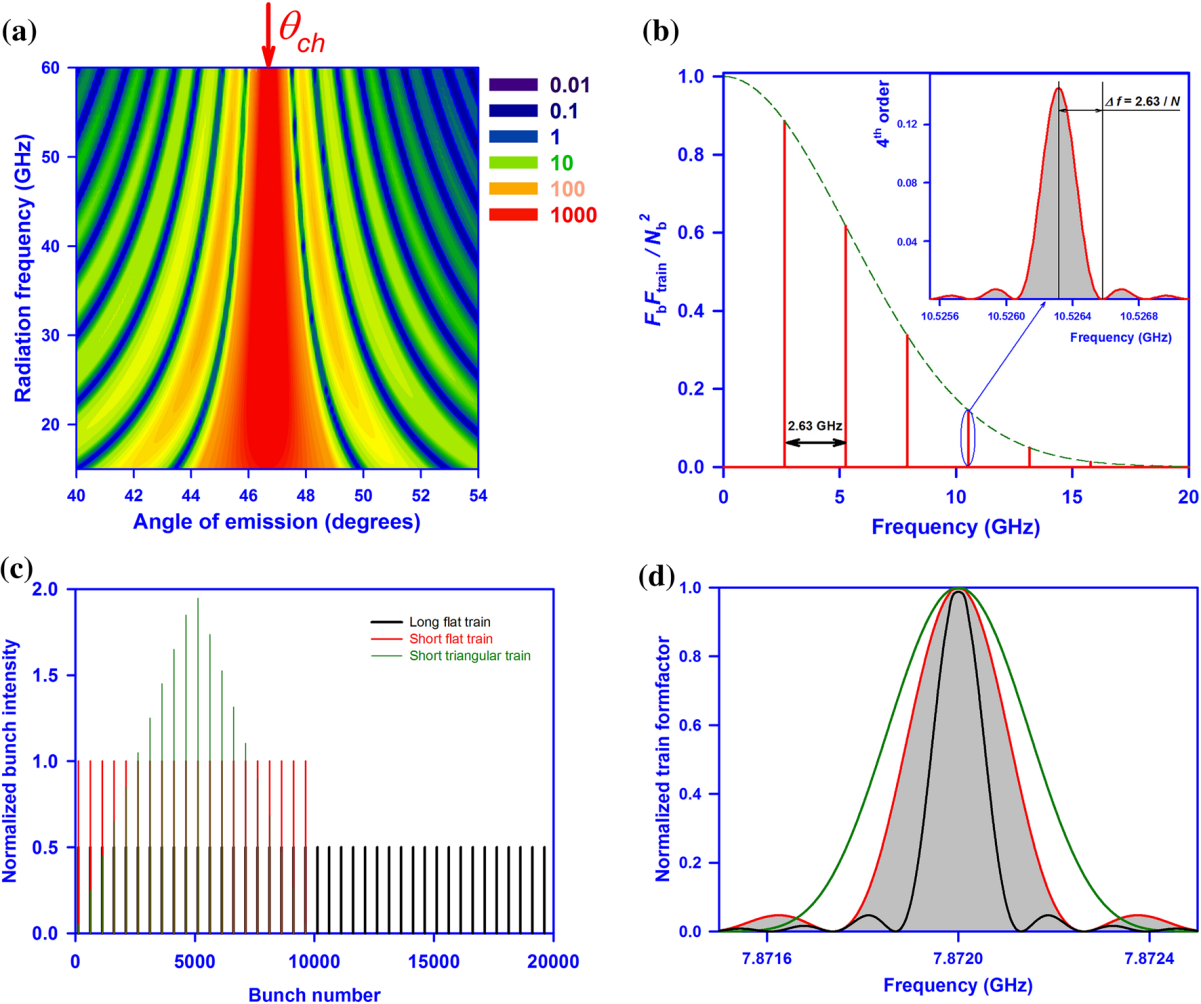
Theoretical calculations. **(a)** The spectral-angular distribution of Cherenkov diffraction radiation calculated for a wavelength of 28.5 mm for a single 6.1 MeV electron passing at an impact parameter of 10 mm away from the Teflon target. The vertical axis is the photon linear frequency *f* = *ω*/2π. The horizontal axis is the polar observation angle. The maximum of the angular distribution corresponds to the Cherenkov angle (*θ*_ch_ = 46.5^0^ shown with a red arrow) defined by Eq. (1) and refracted due to Snell’s law (for Teflon *n* = 1.45 in the frequency range of interest); **(b)** The red line is the normalized form factor of coherent super-radiant Cherenkov diffraction radiation generated by a train of *N*_b_ = 10^4^ bunches (6.3 mm long) spaced by 114 mm. The green dashed line is the envelope function defined by a single bunch longitudinal dimension Eq. (4). The train generates a series of monochromatic lines starting from a fundamental frequency of *f*_1_ = 2.63 GHz representing the frequency of the accelerating cavity used to boost the energy into the electron beam. The lines are spaced in 2.63 GHz intervals and the intensity goes down for higher harmonics due to the decrease in coherency of each individual bunch. The insertion in Fig. 2b shows the zoomed in section of the 4th intra-train resonance order. The width of the monochromatic line is defined as a distance from the principal maximum to the first minimum and for each line is given as *Δf*/*f*_k_ = 1/*kN*_b_, where *k* is the intra-train resonance order, or *Δf*/*f*_1_ = 1/*N*_b_. This expression defines the relative monochromaticity of the radiation. **(c)** Three trains of the same total charge with a 2.63 GHz bunch sequence frequency: green – short triangular train, red – short flat train, and black – long flat train. **(d)** The third diffraction order of the Fourier transforms of corresponding trains from Fig. 2c. A longer flat train generates monochromatic radiation with narrower bandwidth.

As a target we considered a triangular Teflon prism. The radiation generated inside the prism, propagates towards the outer interface, and refracts out towards the observation point (see Fig. 1). ChDR spectral-angular distribution is illustrated in Fig. 2a. The Cherenkov angle for n = 1.45 is *θ*_ch_ = 46.5^0^ which coincides with the maximum emission angle, as the output interface is parallel to the ChDR wavefront. The diffraction fringes due to finite outer target dimensions justify the word “diffraction” in the new name.

The coherent radiation spectrum is illustrated in Fig. 2b. The green dashed line represents an envelope function of Eq. (4) defined by a single bunch length. The shorter the bunch is, the broader the coherent radiation spectrum is. However, the spectrum is not uniform, but consists of a number of equally spaced lines. Each line shape is dictated by the train form factor in Eq. (5). The width of this distribution is defined by the distance from the principal maximum to the first diffraction minimum in the spectrum (see insertion in Fig. 2b), i.e.

6 $$\frac{\Delta f}{f} = \frac{\Delta \omega }{\omega } = \frac{1}{{kN_{{\text{b}}} }}$$

The theory described above is idealised assuming perfect bunch-by-bunch coherency and identity. In the case of 10^4^ bunches the relative bandwidth of the intra-train resonance is 10^–4^/*k*, where *k* is the resonance order. For bunch frequency *f*_b_ = 2.63 GHz the distance between the first and the last bunch is 1.14 km, i.e. when the first bunch generates radiation the last one is still in the cathode. The question is: how narrow a bandwidth can we generate?

Initially the super-radiant emission was observed at a CW electron beam of a Scanning Electron Microscope^25^. The beam was moving parallel to a diffraction grating (Smith-Purcell radiation (SPR) geometry^6^). The field induced at the grating acted back on the beam forcing it to microbunch. A serious enhancement of the Smith-Purcell radiation has been observed. Since then, advanced Orotron technology has been developed to generate the sub-THz high power radiation beams^26,27^. However, this technology has strong threshold requirements on the beam current and the transverse electron beam quality, limiting its use for multi-THz photon generation. An obvious solution was to utilise an initially pre-bunched beam available in almost all high energy accelerators. Therefore, there were no problems generating radiation, however it was difficult to observe the radiation spectrum with high enough spectral resolution. A super-radiant regime was observed at Canadian light source using synchrotron radiation mechanism with a very high resolution Fourier transform spectrometer^28^ and in ANKA light source in Germany^29^, however, the width of the observed lines was precisely defined by the spectrometer resolution. Recent advances in high frequency electronics drastically improved the situation. The SPR enhancement has been observed at the 15 MeV pre-bunched electron beam^30^. As a detection system the authors used a heterodyne receiver. The number of bunches generated was 1500, however the measured line width revealed only 550 bunches. Moreover, the shape of the lines was significantly distorted. Therefore, either the heterodyne receiver limit was reached, or the train is seriously non-uniform. Figure 2c illustrates three different train configurations with their Fourier transforms shown in Fig. 2d (3rd resonance order). A better monochromaticity for the same total charge can be achieved by increasing the train length. If the train is significantly non-uniform, e.g. possesses a triangular shape with the train normalized form factor being

7 $$G_{{{\text{train}}}}^{\Delta } \left( {\omega ,f_{{\text{b}}} } \right) = \left( {\frac{2}{{N_{b} }}\frac{{{\text{sin}}\left( {\frac{{N_{{\text{b}}} \omega }}{{4f_{{\text{b}}} }}} \right)}}{{{\text{sin}}\left( {\frac{\omega }{{4f_{{\text{b}}} }}} \right)}}} \right)^{4}$$

the line width can be broadened as well (see Fig. 2d).

In our experiment we used the state-of-the-art measurement equipment in order to demonstrate that ultra-monochromatic radiation can still be generated non-invasively via the novel ChDR process and can be described by classical theory, even for very long trains.

## Results

The experiment was performed at the Tomsk 6.1 MeV microtron (see “Methods”). A schematic drawing is illustrated in Fig. 1. A beam of relativistic particles grouped in bunches passes by a Teflon prism installed at an impact parameter of 10 mm away from the beam trajectory. Since the shortest wavelength measured was 15 mm, the distance of 10 mm is as good as zero, because it is much shorter than the electron field radius defined by Eq. (2). On the other hand, as the rms beam size is about 1 mm, the contribution from classical Cherenkov radiation, due to direct interaction of the beam halo with the target, is negligibly small.

**Figure 3:**
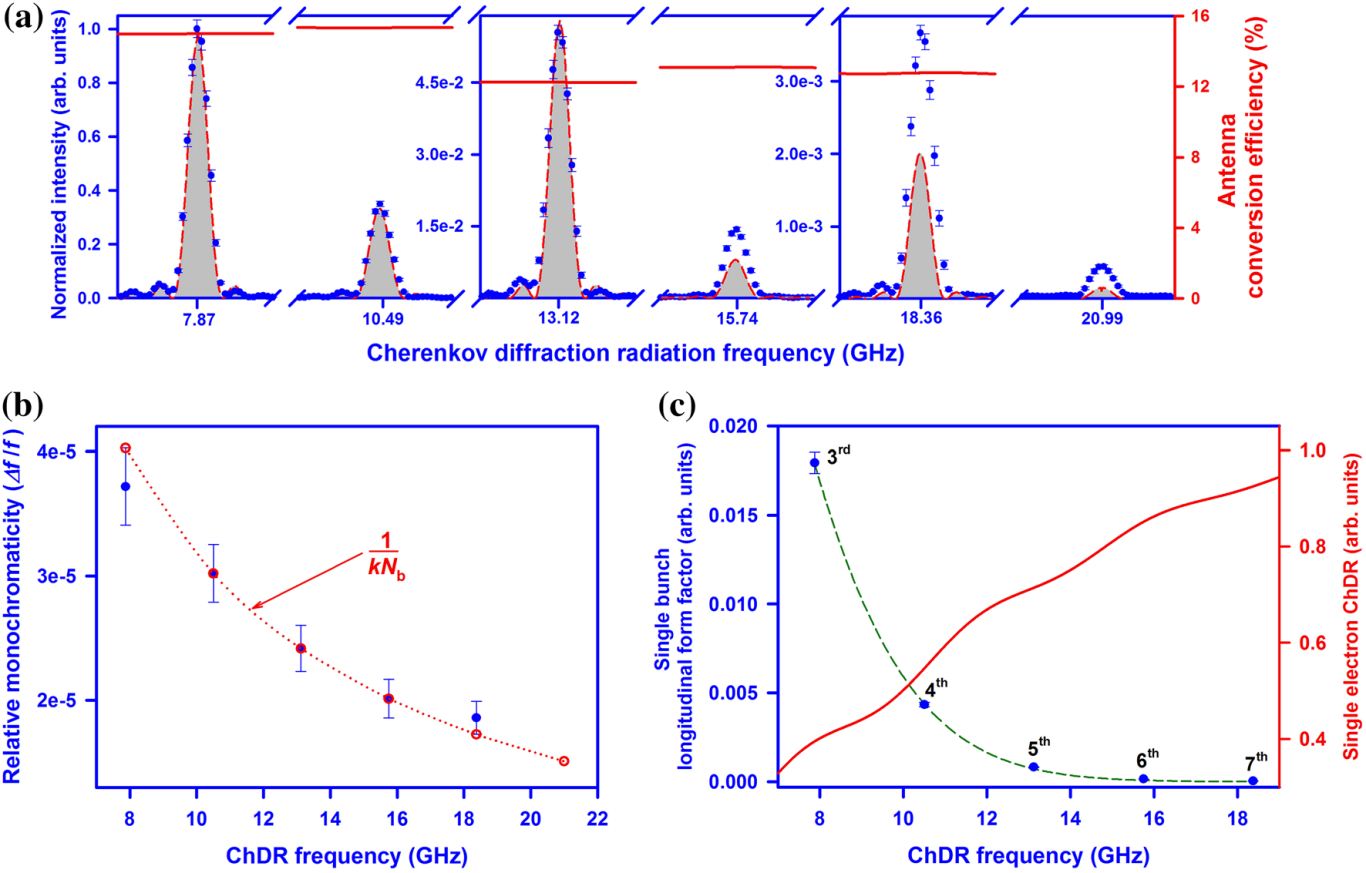
| Experimental results. (**a**) Six diffraction orders (from 3rd to 8th) of the intra-bunch resonance generated via the Cherenkov diffraction radiation mechanism. The horizontal axis is the photon linear frequency *f* = *ω*/2π. The intensity is normalised to the maximum intensity of the 3^rd^ resonance order. The shadowed areas under red dashed lines represent the calculations performed using the polarization current approach^24^ and Eq. (3). The red solid lines represent the conversion efficiency of the horn antenna, measured using a Vector Network Analyser (see “Methods”). **(b)** Relative monochromaticity as a function of the coherent ChDR frequency. The dashed line represents the classic hyperbolic dependence of 1/*kN*_b_. **(c)** The red line represents a single electron ChDR spectrum^24^; Blue dots represent a single electron bunch form factor – this was obtained from the experimental results, i.e. the maxima of each resonance order were normalised to the antenna wavelength efficiency and the single electron spectrum. The form factor was fit with an exponential expression (Eq. 4) to estimate the root-mean-square Gaussian single bunch length, extracted to be 8.16 ± 0.05 mm. The fit is shown by the green dashed line.

The detection system consisted of a multi-band horn antenna (6.5–18 GHz). Technically, frequencies above 18 GHz can also be detected at a significantly reduced efficiency. The antenna was plugged into a 50 GHz spectrum analyser (see “Methods”). The spectra of the 3rd–8th harmonics of super-radiant coherent ChDR are shown in Fig. 3a. The red dashed lines show the results of analytical calculations using Eq. (3). The spectra were normalized to the intensity of the 3rd harmonic to compare with the theory. The radiation peak power at 7.87 GHz was *P*_peak_ = 0.46 μW with the average power generated by the microtron being P_ave_ = 15 pW, which is of the same order of magnitude as the peak power in a synchrotron^31^.

The analytical calculation nearly perfectly matches the shape of the experimental observation, including subsidiary diffraction maxima on both sides of each principal peak (see Fig. 3a). The classical width determined by Eq. (6) and directly extracted from the results was Δ*f* = 317 ± 8 kHz. The relative monochromaticity is demonstrated in Fig. 3b which follows the Eq. (6). The number of bunches contributing to the peak formation was *N*_b_ = 8297 ± 202, corresponding to the train duration of 3.16 ± 0.08 μs, which is consistent with the input power duration signal measured with the spectrum analyser. Despite a very long train length, a perfect coherency was observed.

The experimental intensity decreases slower than the theoretically calculated one because the longitudinal bunch shape is not really Gaussian. Even if the core of the bunch can be approximated by a Gaussian distribution, the tails are very complicated, vary from accelerator to accelerator and are defined by constructive uncertainties and peculiarities. Nevertheless, from the experimental data we can deduce a Gaussian equivalent rms bunch length^32^. The form factor defined by Eq. (4) was obtained from the experimental results (Fig. 3a, blue dots). The maxima of each peak were normalised by the squared beam charge, the antenna efficiency (Fig. 3a, red curve) and the single electron spectrum intensity (Fig. 3c, red curve). The obtained results (Fig. 3c, blue dots) were fit with Eq. (4) and added to the Fig. 3c as a green dashed line. The rms Gaussian bunch length was extracted to be 8.16 ± 0.05 mm. This is the most precise bunch length measurement performed at this facility.

## Discussion

### Light sources

These days 3rd generation (synchrotron radiation storage rings) and 4th generation (linear accelerator free electron lasers) light sources represent the state-of-the-art in photon generation. The storage rings implementing low alpha mode^28,31^ or utilizing microbunch instabilities generate intense THz radiation beams^33^. The bunch train can be treated as continuous. Therefore, the intra-train resonance line width is defined by phase noise, which is very narrow, as was demonstrated at the SOLEIL facility^31^. Nevertheless, the flexibility of the source is very limited.

At synchrotron light sources, the position of monochromatic maxima in the spectrum is fixed and determined by the accelerating field frequency and the ring circumference. If the response bandwidth of a biological or chemical sample is comparable to or smaller than the fundamental frequency, the sample might leave a distorted or even no signature in the THz radiation spectrum, leading to misunderstanding or wrong conclusions. On the other hand, the generation of radiation frequencies above 1 THz, stably and reliably, is practically impossible in circular machine because of complicated longitudinal beam dynamics^33^.

In linear accelerator based free electron lasers the light is generated by a very long train of extremely short bunches. In that case, the generation of very highly monochromatic multi-THz radiation beams is possible. However, these facilities are optimized to generate intense X-ray laser radiation^34^. They implement multistage ultra-precise synchronization techniques for stable emission as required by the user community. Therefore, the bunch sequence frequency is also fixed and defined by the accelerating field frequency.

In order to make these facilities useful in the THz frequency range^33–35^, an ultra-precise heterodyne detection system is needed, which is not widely available yet in the multi-THz frequency range.

### Compact linear accelerator facilities

Small and compact accelerators with energies from a few tens to a few hundreds of MeV such as LUCX^36^, CLEAR^32^, or CLARA^37^, can be an alternative to central Light Source facilities. The bunch sequence frequency can be easily tuned within a range of ± 20 MHz at a 3 GHz RF acceleration field. At the 75th harmonic corresponding to 225 GHz and above, the monochromatic line will shift over a range of ± 1.5 GHz covering the entire frequency range. Any sample placed on the transmission line can be scanned across the frequency range to determine any peculiarity in the absorption or reflection spectrum with the resolution of the line bandwidth defined by the train length. A conventional Fourier transform spectrometer (e.g. Martin-Puplett or Michelson interferometer) can be used to monitor whether the line is transmitted or not. It is very important that this methodology can be realised at multi-THz frequencies as well. The non-invasive nature of the phenomenon enables facilities to build multiple beamlines in a single linear accelerator providing opportunities for different uses in a multi-user community.

### Monochromaticity of X-ray free electron lasers (FEL)

In X-ray FELs, the coherency is achieved via the SASE (Self Amplified Spontaneous Emission) process^35^. An extremely short electron bunch is needed to minimize the time required to complete the SASE microbunching and achieve saturation in X-ray emission. However, the X-ray monochromaticity also depends on the bunch length. Figure 2b,c illustrate that the longer the train is, the narrower the spectral line is. Therefore, by optimizing the bunch length versus SASE efficiency, X-ray FELs might generate significantly higher quality X-ray beams.

## Summary

We have demonstrated that a long dielectric radiator can be used to generate an intense electromagnetic beam via the novel coherent ChDR process. A long train of bunches can generate highly monochromatic radiation, in which the relative bandwidth follows the 1/*kN*_b_ dependence. The shortest coherent wavelength is determined by a single bunch duration that can reach sub-100 fs in modern accelerators and supply multi-THz frequencies. In a compact linear accelerator the bunch frequency can be varied with high accuracy, enabling it to scan across the entire frequency range, opening doors for ultra-precise THz spectroscopy. The monochromaticity of the emitted radiation can be optimised by shaping the longitudinal particle distribution in the train.

## Methods

### Accelerator facility

A 6.1 MeV electron microtron was used for the experiment^22,23^. The purpose of this machine is to provide a test bench for experiments related to the interaction of relativistic electrons with matter. The electron beam is generated by a thermionic gun and is captured by a 2.63 GHz accelerating RF (radio-frequency) field. The beam moves in a circular orbit making 12 turns and each time gaining 0.511 MeV energy until extraction. During the acceleration process the energy tail of the bunches is cleared and a 3.2 μs long train consisting of about 10^4^ bunches (which are 8.2 mm long) is formed. A bending magnet leads the beam towards the in-air experimental area. The normalized beam emittance was about 4 π mm mrad. Using two pairs of quadrupole magnets (quadrupole doublets) enabled us to shape the beam in the vicinity of the target making it quasi-parallel and as small as 1 mm rms (root-mean-square) in the transverse dimension. The repetition rate is 10 Hz. After the experimental area, the beam was terminated in a Faraday cup, measuring the beam total charge. Each bunch contained on average 10^8^ particles adding up to 10^12^ particles per train.

### Detection system

The generation of ultra-highly monochromatic radiation is only half of the challenge. The other half is to observe it. Detection electronics have been intensively developed over the last few decades and in several frequency ranges, measurements with sufficient spectral resolution have now become possible. In our experiment, the radiation initially received by a horn antenna (A-info Ltd., LB-65180–15-C-SF, 6.5–18 GHz and aperture 78 mm in width and 65.9 mm in height) induced currents on the pin inside the antenna waveguide. The amplitude and frequency content of the current is directly proportional to the radiation characteristics. The current was fed into the high speed frequency analyser (Keysight Technologies, N9040B) with the lowest noise level of -171 dBm and the highest fractional frequency resolution of 10^–11^. Since the span window of the spectrum analyser is rather narrow (100 MHz), we could only monitor a single monochromatic line at a time. The spectral efficiency of the antenna has been tested with a Vector Network Analyser (Rohde & Schwarz ZVB20) and found to be -8 dB.

### Power measurements

The signal strength for the 3rd intra-train resonance harmonic is − 40 dBm. Taking into account the − 8 dB conversion efficiency of the antenna and the fact that the measurements were done over a 100 μs, while the train length was only 3.2 μs, the measured peak power is P_peak_ = 0.46 μW at 7.87 GHz. Due to a very low duty cycle (Q = 3.2⋅10^–5^), the average power is P_ave_ = 15 pW which is the same order of magnitude as in a synchrotron^31^ for a given antenna aperture. However, the total integrated power measured by the horn antenna across all 6 resonance orders was only a factor of 10 larger, which is several orders of magnitude lower than in a synchrotron. This is because the spectrum is much broader and extends to THz frequencies, and the train is practically continuous.

### Analytical theory

To calculate the single electron spectrum we have used the polarization current approach^24^. This approach merges together different mechanisms of polarization radiation. However, it also has approximations, e.g. the target is infinite in the vertical direction and the detector is infinitely far from the radiator (far-field approximation). Because of that we are not able to compare absolute intensity with the experiment. Nevertheless, the single electron radiation spectrum is a slow function of frequency, enabling us to use this approach to compare the shape of the resonance maxima.
